# Hypoglossal nerve stimulation for obstructive sleep apnea: updated position paper of the German Society of Oto-Rhino-Laryngology, Head and Neck Surgery

**DOI:** 10.1007/s00405-021-06902-6

**Published:** 2021-06-21

**Authors:** Armin Steffen, Clemens Heiser, Wolfgang Galetke, Simon-Dominik Herkenrath, Joachim T. Maurer, Eck Günther, Boris A. Stuck, Holger Woehrle, Jan Löhler, Winfried Randerath

**Affiliations:** 1 Department for Otorhinolaryngology, University of Lübeck, Ratzeburger Allee 120, 23538 Lübeck, Germany; 2 Sleep Medicine Work Group of the German Society of Oto-Rhino-Laryngology, Head and Neck Surgery (DGHNO), Bonn, Germany; 3 Work Group Sleep Surgery of the German Society for Sleep Research and Medicine (DGSM), Schwalmstadt-Treysa, Germany; 4 Work Group Apnea of the German Society for Sleep Research and Medicine (DGSM) (DGSM), Schwalmstadt-Treysa, Germany; 5 Section 8 Sleep Medicine of the German Respiratory Society (DGP), Berlin, Germany; 6 German Professional Association of Pulmonologists, (BdP), Heidenheim, Germany; 7 German Professional Association of Ear, Nose, Throat-Physicians (BVHNO), Neumünster, Germany; 8 Department for Otorhinolaryngology, Technical University Munich, Munich, Germany; 9 Department for Pulmonolgy, VAMED Klinik Hagen-Ambrock, Hagen, Germany; 10 Institute of Pneumology at the University of Cologne, Bethanien Hospital, Clinic for Pneumology and Allergology, Centre of Sleep Medicine and Respiratory Care, Solingen, Germany; 11 Department of Otorhinolaryngology, Head and Neck Surgery, Sleep Disorders Center, University Hospital Mannheim, Mannheim, Germany; 12 Practice for Otorrhinolaryngology, Stuttgart, Germany; 13 Department of Otolaryngology/Head & Neck Surgery, University Hospital Giessen and Marburg, Philipps-Universität Marburg, Marburg, Germany; 14 Sleep and Ventilation Center Blaubeuren, Lung Center Ulm, Ulm, Germany; 15 ENT Clinic, Maienbeeck, Bad Bramstedt, Germany

**Keywords:** Hypoglossal nerve stimulation, Obstructive sleep apnea, Neurostimulation, CPAP failure, Sleep endoscopy, DISE

## Abstract

Since the first statement of the German Society of Oto-Rhino-Laryngology, hypoglossal nerve stimulation (HNS) is meanwhile an established treatment option for obstructive sleep apnea (OSA). There are three HNS systems available in Germany which differ in their technical details of the underlying comparable basic principle. For the unilateral HNS with respiratory sensing, several comparative studies, high-volume register analysis and long-term reports exist. The continuous HNS without respiratory sensing does not require a sleep endoscopy for indication. For the bilateral continuous HNS as the single partially implantable device, a feasibility study exists. For indication, the assessment of positive airway pressure failure by sleep medicine is crucial, and the decision for HNS should be made in discussion of other treatment options for at least moderate OSA. The implantation center holds primarily responsibility among the interdisciplinary sleep team and is primary contact for the patient in problems. This depicts why structural processes are required to secure outcome quality and minimize the complications. The aftercare of HNS patients can be provided interdisciplinary and by different medical institutions, whereat, minimal reporting standards to document outcome and usage are recommended.

## Introduction

Since the first position paper of the sleep medicine work group of the German Society of Oto-Rhino-Laryngology, Head and Neck Surgery (DGHNO) [1], hypoglossal nerve stimulation (HNS) attained increasing attention in the treatment of obstructive sleep apnea (OSA). Within recent years, there is accumulating evidence on patient selection, surgical techniques, treatment pathways, long-term data as well as experiences with special patient groups. With this positional paper, the implementation of HNS in treatment pathways of the German healthcare systems shall be highlighted. The position paper was established and finally approved by several sleep medicine related societies on November 27th 2020: The sleep medicine work group of the DGHNO, the work group apnea and sleep surgery of the German society for sleep research and medicine, the German Professional Association of pulmonologists, the German Professional Association of Ear, Nose, and Throat-physicians, and the German Respiratory Society [2].

## Aims

This positional paper aims to

- Warrant a high quality of interdisciplinary patient care regardless of residence in Germany and socio-economic status,
- Provide a structural, procedural, and outcome-based interdisciplinary care pathway in the provision of hypoglossal nerve stimulation,
- Implement evidence-based decision making,
- Minimize therapy risks and adverse effects,
- Avoid OSA-associated complications and co-morbidities as well as to improve quality of life among patients with non-adherence to positive airway pressure therapy (PAP).

## Available hypoglossal nerve stimulation systems

Currently, there are three hypoglossal nerve stimulation systems for OSA treatment available in Germany. For a more detailed description of the underlying technology, there are extensive descriptions and reviews [3, 4]. The Federal Joint Committee (G-BA) decided in their assessment from March 5th 2020 that “neither the underlying principle (2.4.1.2) nor the indication (2.4.1.3) differ relevantly between the assessed treatment method with regard to the established inpatient treatment method; thereby, the here assessed treatment method (*here, the system provided by Nyxoah, Inc.—added information by the authors*) reveals no novel theoretical–academic concept” [5].

### Selective hypoglossal nerve stimulation with respiratory sensing (Inspire Medical Systems, Inc.)

Selective hypoglossal nerve stimulation with respiratory sensing uses an intercostal sensor for the detection of the patient’s breathing cycle. This information is processed in the subclavian impulse generator, which then delivers an impulse to the stimulation electrode at the lateral neck. The submentally positioned cuff of the electrode activates protrusor branches of the distal hypoglossal nerve and herewith, opens the upper airway. The system received CE marking in October 2010, FDA approval in April 2014, and accreditation by the Japanese health authority PMDA in June 2018.

In an early study [6], a complete concentric collapse (CCC) at velum level during drug-induced sleep endoscopy (DISE) was associated with a highly increased non-response rate to treatment with HNS. With a prevalence of 20–25% among PAP intolerant patients [7, 8], such CCC is frequent and needs to be ruled out in a DISE. There was a good response rate to HNS in a study with randomized therapy withdrawal [8] for patients with a body mass index (BMI) below 32 kg/m^2^ and an apnea hypopnea index between 20 and 50 events per hour. Data from national and international, multicenter and single-center cohort and register studies revealed that inclusion criteria could be broadened (AHI between 15 and 65/h, BMI up to 35 kg/m^2^) without risking lower response rates and lower therapy adherence [9–12]. In a comparison of response rates, patients with a BMI below 32 kg/m^2^ did not differ from patients with a BMI between 32 and 35kg/m^2^ [13]. In publications of the international ADHERE register, the rate of unwanted events and complications is about 2–6% [10].

For optimal positioning of the stimulation electrode, neuromonitoring guidance is recommended to identify the protrusor branches of the hypoglossal nerve [14–16]. The system is activated four weeks after implantation with another four weeks to allow acclimatization. About eight weeks after implantation, a polysomnographic therapy adjustment is performed in a sleep laboratory to optimize OSA therapy. Later, a regular annual follow-up appointment is recommended, including objective therapy control with a home-sleep testing device (HST). If needed, the polysomnographic adjustment is repeated. The impulse generator contains a lithium battery with an approximated lifetime of about 10 years.

### Continuous hypoglossal nerve stimulation (LivaNova, Inc., former ImThera Medical)

Continuous hypoglossal nerve stimulation does not require respiratory sensing. Six electrodes are located in a ring-like stimulation cuff around the main hypoglossal nerve and alternately stimulate the targeted nerve branches by changing electric fields. The impulse generator is located in the subclavian area and contains an accumulator, which needs to be charged regularly. The lifespan of the rechargeable battery is estimated to last about 15 years. Four–six weeks after implantation, the most suitable electrode combination and amplitude to achieve a stabilization of the upper airway are calculated in a polysomnographic-assisted study. In contrast to selective HNS with respiratory sensing, a DISE is not necessary for the indication [17, 18]. The aura6000 system received CE marking in March 2012. Since 2015, the FDA approval study (“THN3”) is still running, but is no longer actively recruiting participants.

### Bilateral continuous hypoglossal nerve stimulation (Nyxoah S.A.)

In contrast to both systems described before, the Genio system (Nyxoah S.A.) is a submentally placed HNS with wing-like electrodes close to both hypoglossal nerves [19]. The distal placements allow an activation of more protrusor branches and opens the airway by tongue protrusion similar to selective HNS. The preset processing unit with a rechargeable battery transcutaneously delivers the required energy to the implant during sleep. The stimulation is phasic and asynchronous and is adjusted in a polysomnography similar the continuous HNS. The external processing center and accumulator is charged during daytime. A DISE is necessary to rule out a complete concentric collapse at velum level. The Genio system received CE marking in March 2019. The FDA approval study (“DREAM”) started in September 2020.

## Overview of evidence

As all available systems differ in technical details, mode of operations, and market approval, the review of evidence has to be differentiated.

For the HNS with respiratory sensing, about 100 publications exist and cover a long-term view up to 60 months. For market approval, the STAR study evaluated the safety and effectiveness of the HNS in Europe and the US during 2010 and 2013. This study covering a selected cohort of 126 patients with moderate to severe OSA has the highest evidence level, as it contains a randomized therapy withdrawal arm of therapy responders. Data on annual follow-up reports exist up to 5 years [8, 20–22]. Currently, a randomized controlled study with therapy withdrawal of 89 patients regardless of treatment responsiveness is under review (“EFFECT”). This study assessed the recurrence of daytime sleepiness and OSA events under subtherapeutic voltage [23]. There are several multicenter cohort studies which confirmed safety and effectiveness of daily routine use [9, 24–26], including 2- and 3-years of follow-up data [25]. In addition, the outcomes of monocentric 4-year follow-up results can also be found in the literature [26]. In therapy responders, HNS with respiratory sensing can normalize sleep-related quality of life with very good patient satisfaction [27, 28]. Patient age and previous upper airway surgery appear not to affect effectiveness [29, 30]. Several retrospective comparative studies show better OSA outcomes of HNS treatment compared to traditional sleep surgery [31–33]. First reports documented positive effects on glucose metabolism and heart frequency variability [34, 35]. A recent prospective multicenter study, which show relevant improvement of OSA in HNS therapy compared with no treatment [12].

For the continuous HNS ImThera system, there are first studies for safety and effectiveness which reveal an improvement in OSA [17, 36]. In the next months, the results of the European and US randomized controlled study with 135 patients are expected to be published (“THN3”).

So far, Nyxoah’s Genio system was assessed in two smaller case series during device development. There is a publication on 27 patients with a 6-month follow-up published in 2019, which demonstrated the benefit of this system [19].

## Indication

- Assessment of PAP-nonadherence

Careful candidate selection is essential for good HNS outcomes. Currently, HNS is recommended as a second line treatment after PAP therapy. Therefore, besides suitable evaluation regarding any HNS, the reasons for PAP failure need to be documented during the screening process [37]. It is, therefore, advisable to check for PAP optimization options and to evaluate other OSA treatment options in the individual decision-making process.

b. Interdisciplinary decision-making is based on implanting center’s authority/responsibility

The interdisciplinary patient care pathways with HNS are usually collaboratively lead by the departments of otorhinolaryngology and sleep medicine. A standardized treatment pathway and a good communication between specialties are essential to achieve good OSA control and effective usage of HNS. Therefore, several requirements should be fulfilled by the HNS team (Table 1, see chapter 6). Ideally, patient selection, DISE, implantation, therapy adjustment and control are carried out in a department of otorhinolaryngology with profound sleep medicine knowledge or in a sleep medicine center under surveillance of the implanting surgeon. The HNS therapy can be provided by teams across different locations, as long as the responsibilities and communication have been clarified and regular team conferences on patient pathways take place. It has to be highlighted that the implanting center plays a central role in the responsibility for patient selection and therapy adjustment and should be capable of evaluating sleep medicine reports and assessing the documentation of PAP nonadherence.

**Table 1:** Structural requirements in performing the treatment

Surgical team, experienced in head neck surgery and a 24-h on-call duty
Sleep medicine team, experienced in patient care with obstructive sleep apnea
Proficient experience in endoscopic evaluation of the upper airway, especially of nasopharynx, velopharynx, oropharynx including tonsils, tongue base, hypopharynx, and epiglottis
Knowledge in the assessment and interpretation of sleep diagnostics, opportunity to perform home sleep tests or polysomnography, where applicable, in multidisciplinary cooperation
Knowledge in performing drug-induced sleep endoscopy, in particular on the evaluation of HNS suitability
Performing the surgical implantation using optical magnification and intraoperative neuromonitoring after training and certification by the manufacturer
Opportunity to provide adequate postoperative care in patients with moderate to severe obstructive sleep apnea, low-threshold access to intermediate care units and/or intensive care units
Activation and titration of patients together with qualified technical assistants
Provide resources for long-term follow-up of HNS and if necessary, in corporation with external sleep medicine physician
Experience in transnasal endoscopy for postoperative HNS therapy optimization
Timely availability for HNS patients with requests
Implanting center managed preferably by otorhinolaryngologists or head neck surgeons with sleep medicine qualification/board certification

*HNS* hypoglossal nerve stimulation

The DISE is relevant for whether a patient is suitable for PAP alternatives, especially regarding HNS with respiratory sensing [6, 7]. During this investigation, the exclusion criteria of complete concentric collapse at velum level and other treatment options can be assessed or ruled out [37]. Therefore, profound expertise is needed to perform and evaluate a DISE. There is respective literature about DISE technique and requirements [38, 39].

In case of external referrals, standards are necessary for preoperative diagnostics and their ongoing quality conformance. Therapy adjustment should only be performed outside the implanting center with great care, to ensure that the recommended and correct treatment pathway is followed. In particular, in case of suspected technical issues, the implanting centers are mainly responsible for the therapy and should be addressed immediately. Therapy outcomes should be assessed in joint conferences with all partners.

Informed consent for the implantation needs certified forms and should be completed by a specialist of the implanting center.

c. Guidelines of the German society for sleep research and medicine

Recently in 2020, the guidelines for the treatment of sleep-related breathing have been updated under guidance of the German society for sleep research and medicine, DGSM. Using a structured literature review, this S3-leveled guideline recommends—with its primary focus on HNS with respiratory sensing—that HNS should be considered in patients with moderate to severe OSA (AHI between 15 and 65 events per hour), PAP intolerance or inefficiency, in the absence of Class II or higher obesity (BMI below 35 kg/m^2^), and in patients without anatomical obstructions in the upper airway (evidence level 1b, recommendation level B, concordant strong consensus) [40].

d. Clinical practice

In Germany, there are more than 35 implanting centers for HNS with specialized infrastructure and certification by the manufacturer. HNS is considered in patients using the following criteria:

- Apnea hypopnea index between 15 and 65 events per hour,
- Body mass index ≤ 35 kg/m^2^,
- The portion of central events is ≤ 25% in relation to the entire night’s AHI,
- The exclusion of a complete concentric collapse at the velum level during DISE When bilateral HNS or HNS with respiratory sensing is considered,
- PAP-nonadherence or non-compliance (chapter 4),
- The absence of neuromuscular diseases and
- Appropriate motivation of the patient.]

## Quality requirements in a hypoglossal nerve stimulation center

Hypoglossal nerve stimulation therapy should be allocated in centers with sleep medicine expertise and specialized infrastructure. The treatment pathway and responsibilities should be defined when the center is established and need regular review.

- Structural requirements for the treatment

The following structural requirements need to be fulfilled in implanting centers. This assures a smoother process, better outcome quality and helps to minimize complications (Table 1).

b. Follow-up of patients with hypoglossal nerve stimulation

It is very important to provide long-term follow-up resources for patients with HNS. The patient care and follow-up can be carried out in the implanting center or by external physicians with sleep medicine qualification. The implanting centers need to provide timely availability for potential medical and technical complications. Special consultation hours help to provide appropriate service for HNS candidates and patients. If the follow-up is provided by external sleep physicians, the implanting center should be updated about the patients' treatment course for quality management (see chapter 6).

## Quality management and supply research

For assurance of therapy quality, implanting centers should document therapy effects properly and do regular evaluations (Table 2). Industry driven registries and independent assessments are valuable and should cover data security concerns. As minimal requirements, patient aspects, therapy effects and usage should be recorded too.

**Table 2:** Recommended variables regarding patient’s aspects, therapy effects and usage

Variable	Baseline	Implantation	Early follow-up	Annual follow-up
Demographic aspects (age, gender, height, weight)	X		X	X
Apnea hypopnea index (AHI)	X		X	X
Oxygen desaturation-index (ODI 4%)	X		X	X
Epworth Sleepiness Scale (ESS)	X		X	X
Side effects and complications		X	X	X
Therapy adherence (usage per night)			X	X

In recent years, OSA treatment with HNS is well-described as a second line therapy in PAP failure and positioned in national guidelines. All three available HNS systems differ in technical details of the common underlying therapy principle, indication and, most of all, evidence. The implanting centers obtains the main responsibility within the interdisciplinary treatment concept. Quality management is essential for the entire treatment pathway, especially for the long-term follow-up.
